# Bovine Milk Oligosaccharides with Sialyllactose Improves Cognition in Preterm Pigs

**DOI:** 10.3390/nu11061335

**Published:** 2019-06-14

**Authors:** Karina Obelitz-Ryom, Stine Brandt Bering, Silja Hvid Overgaard, Simon Fristed Eskildsen, Steffen Ringgaard, Jonas Lynge Olesen, Kerstin Skovgaard, Stanislava Pankratova, Bing Wang, Anders Brunse, Anne Birgitte Heckmann, Martin Peter Rydal, Per Torp Sangild, Thomas Thymann

**Affiliations:** 1 Comparative Pediatrics and Nutrition, Department of Veterinary and Animal Sciences, University of Copenhagen, 1870 Frederiksberg, Denmark; karina.ryom@sund.ku.dk (K.O.-R.); sbb@sund.ku.dk (S.B.B.); siljahvid@msn.com (S.H.O.); stasya@sund.ku.dk (S.P.); anderss@sund.ku.dk (A.B.); martin.rydal@sund.ku.dk (M.P.R.); pts@sund.ku.dk (P.T.S.); 2 Department of Clinical Medicine, Center of Functionally Integrative Neuroscience, Aarhus University, 8000 Aarhus, Denmark; seskildsen@cfin.au.dk (S.F.E.); jonas@phys.au.dk (J.L.O.); 3 Department of Clinical Medicine, The MR Research Centre, Aarhus University, 8200 Aarhus, Denmark; steffen@clin.au.dk; 4 Department of Biotechnology and Biomedicine, The Technical University of Denmark, 2800 Lyngby, Denmark; kesk@dtu.dk; 5 Department of Pediatrics and Adolescent Medicine, Rigshospitalet, 2100 Copenhagen, Denmark; 6 Laboratory of Neural Plasticity, Department of Neuroscience, University of Copenhagen, 2200 København, Denmark; 7 School of Animal & Veterinary Sciences, Charles Sturt University, Wagga 2678, Australia; biwang@csu.edu.au; 8 Arla Foods Ingredients, 8260 Viby, Denmark; AnneBirgitteLau.Heckmann@arlafoods.com

**Keywords:** sialic acid, cognition, brain, preterm birth, milk

## Abstract

Optimal nutrition is important after preterm birth to facilitate normal brain development. Human milk is rich in sialic acid and preterm infants may benefit from supplementing formula with sialyllactose to support neurodevelopment. Using pigs as models, we hypothesized that sialyllactose supplementation improves brain development after preterm birth. Pigs (of either sex) were delivered by cesarean section at 90% gestation and fed a milk diet supplemented with either an oligosaccharide-enriched whey with sialyllactose (*n* = 20) or lactose (*n* = 20) for 19 days. Cognitive performance was tested in a spatial T-maze. Brains were collected for ex vivo magnetic resonance imaging (MRI), gene expression, and sialic acid measurements. For reference, term piglets (*n* = 14) were artificially reared under identical conditions and compared with vaginally born piglets naturally reared by the sow (*n* = 12). A higher proportion of sialyllactose supplemented preterm pigs reached the T-maze learning criteria relative to control preterm pigs (*p* < 0.05), and approximated the cognition level of term reference pigs (*p* < 0.01). Furthermore, supplemented pigs had upregulated genes related to sialic acid metabolism, myelination, and ganglioside biosynthesis in hippocampus. Sialyllactose supplementation did not lead to higher levels of sialic acid in the hippocampus or change MRI endpoints. Contrary, these parameters were strongly influenced by postconceptional age and postnatal rearing conditions. In conclusion, oligosaccharide-enriched whey with sialyllactose improved spatial cognition, with effects on hippocampal genes related to sialic acid metabolism, myelination, and ganglioside biosynthesis in preterm pigs. Dietary sialic acid enrichment may improve brain development in infants.

## 1. Introduction

More than 10% of all infants worldwide are delivered preterm (gestational age <37 weeks). Improved neonatal care has led to the decreased mortality of preterm infants [1], but morbidities are still common. Several organ systems are affected by preterm birth, including the lungs, gut, and brain, that undergo fast development in the perinatal period [2]. Neurodevelopmental impairment covers a range of brain-related complications involving cognitive, neurologic, and sensory deficits [3]. Preterm infants suffer from more motor and cognitive deficits than term infants, even when assessed at the same postconceptional age. This suggests that the brain is at risk of a maturational deficit due to both shortened gestation and acquired injuries [3]. Among infants born very preterm (gestational age <32 weeks), cognitive deficits are among the most frequently encountered neurological impairments [4,5], and these may last into childhood [6,7].

Nutrition is important for neurodevelopment in preterm infants, but the role of other factors, such as mode of delivery, feeding of human milk versus infant formula, and hospital admission, may play a role. Mother’s own milk is optimal for both preterm and term infants [8], and there seems to be an association between breastfeeding and increased cognitive development in term infants [9,10]. Similarly, improved brain development is documented in breastfed versus formula-fed rhesus macaques [11]. Preterm infants seem to benefit even more from human milk and show improved neurodevelopment compared with formula feeding [12], probably due to higher needs for bioactive milk factors [8].

Human milk oligosaccharides (HMO) have been associated with antimicrobial and immunomodulatory effects and increased delivery of sialic acid for the developing brain [13,14]. Sialic acid is important for gangliosides involved in neural transmission and cognition [15,16]. Human milk is particularly rich in oligosaccharide-bound sialic acid [14], particularly in early lactation. On the contrary, the concentration of sialic acid in infant formulas is significantly lower, and the majority is bound to glycoproteins rather than oligosaccharides [17,18]. Accordingly, the concentration of sialic acid in saliva and brain tissue is higher in breast-fed versus formula-fed infants [19,20,21], but the functional outcome is unknown. Animal studies have investigated the functional brain effects of sialic acid using rats of varying age [22,23,24,25,26,27], mice [28], and full-term neonatal piglets [29]. Sialic acid supplementation has shown to increase the sialic acid concentration in brain gangliosides and improve cognition in term newborn pigs [29,30], but effects in preterm brain development are unknown.

Preterm pigs delivered at 90% gestation show similar immature organ function as observed in preterm infants, including delayed gut and brain development [31,32]. In porcine milk, sialic acid levels also decrease during the lactation period, and the oligosaccharide profile is closer to human than bovine milk [33]. We therefore consider the preterm pig a highly sensitive model for investigating the effects of dietary sialic acid on cognition and neurodevelopment in preterm infants. We investigated structural and functional neurodevelopmental outcomes in preterm pigs with or without supplementation of an oligosaccharide-enriched whey with sialyllactose during the first 19 days after preterm birth. The effect of prematurity per se was tested against term control pigs reared within the same experimental settings. As a term reference group, pigs born naturally and reared by the sow were included, to indicate the neurodevelopmental pattern of normal time of birth and postnatal rearing.

## 2. Materials and Methods

### 2.1. Experimental Design and Animal Procedures

A total of 46 (20 female and 26 male) preterm piglets (Landrace × Yorkshire × Duroc) were delivered by cesarean section from three pregnant sows at 90% gestation (day 106) [31]. The pigs were resuscitated immediately after the cesarean section and placed in oxygenated and temperature-regulated incubators. Within 3 h after birth, a catheter was placed via the transected umbilical artery to allow parenteral nutrition support, and an orogastric feeding tube was placed to allow enteral bolus feeding [32]. The preterm pigs were stratified according to litter and birth weight, and allocated to two groups receiving raw bovine milk supplemented with either an oligosaccharide-enriched whey with sialyllactose (PRE-SAL, *n* = 22) or lactose as control (PRE-CON, *n* = 24). The researchers were blinded to the treatment groups. Passive immunization with maternal plasma, prophylactic and therapeutic oral antibiotic treatment, and iron supplementation were conducted as previously described [34]. The clinical condition of each pig was assessed at least twice daily, and 6 pigs were euthanized during the study period due to clinical complications, resulting in the following final group sizes, PRE-SAL (*n* = 20, 10 female and 10 male) and PRE-CON (*n* = 20, 9 female and 11 male).

To determine the influence of prematurity per se, a litter of fourteen pigs (9 female and 5 male) was delivered by cesarean section as close to the expected full-term as possible, i.e., day 115. They were reared under identical conditions as the preterm pigs and fed the control diet (TERM-CON). As a naturally reared term reference group, another litter of 12 pigs (6 female and 6 male) was born by spontaneous vaginal delivery on day 112. These pigs were reared with their mother under natural rearing conditions and were allowed unlimited natural suckling of the sow (TERM-NAT).

### 2.2. Diets

All artificially reared pigs were fed an enteral diet based on raw bovine milk supplemented with either a bovine milk oligosaccharide-enriched whey with sialyllactose (SAL) or lactose (CON), whereas the vaginal-delivered reference group received mother’s milk only. On the first day, all pigs received minimal enteral nutrition (MEN) at 16 mL/(kg∙day). The amount of enteral nutrition was gradually increased to reach 180 mL/(kg∙day) on day 16. During the first seven days, the pigs were supplemented with parenteral nutrition (PN) at 48–144 mL/(kg∙day) (Kabiven modified with Vamin, Soluvit, Vitalipid, and Peditrace to meet the nutritional needs of preterm piglets [31], all from Fresenius Kabi, Uppsala, Sweden). During the first five days, enteral feeding was administered exclusively through the fitted orogastric tubes. From day five onwards, the pigs were introduced to the milk in drinking troughs and were assisted with the drinking until they mastered the task on their own.

The enteral diet consisted of intact unpasteurized Jersey cow’s milk. The raw milk was stored in aliquots at −20 °C until use. The SAL diet was prepared by adding the SAL ingredient (8.5 g/L oligosaccharide-enriched whey, Lacprodan SAL-10^®^, Arla Foods Ingredients, Viby, Denmark) to the milk, to a final dose of 380 mg sialyllactose per liter of milk. The ingredient contained 4.5% sialyllactose, with a 6:1 ratio of 3’-sialyllactose (3’SL) and 6’-sialyllactose (6’SL), as well as other acidic and neutral bovine milk oligosaccharides (not quantified). To secure isocaloric diets, 6 g of lactose (Variolac^®^ 960, Arla Foods Ingredients) were added per liter of milk in the CON diet. The diets were prepared on a daily basis and stored at 4 °C until use. Aliquots were heated to a maximum temperature of 40 °C prior to feeding. Siallyllactose concentrations in the milk, including 3’SL and 6’SL, were determined by high-performance anion exchange chromatography with pulsed amperometric detection (HPAEC-PAD). The total levels were 433 ± 5 mg/L and 120 ± 2 mg/L in the SAL and CON diets, respectively, with a ratio of 3’SL and 6’SL of 5 to 1.

### 2.3. Motor Acquisition, Home Cage Activity, Open Field Assessment, and Cognitive Performance

During the first days after birth, the pigs were monitored closely, and the time to first eye opening, first stand, and first walk, were recorded. Likewise, the time spent to learn to drink full milk boluses from the trough was recorded. Continuous recordings of home cage activity were obtained for each pig on days 2–9 and analyzed with the software PIGLwin (Ellegaard Systems, Faaborg, Denmark) [32]. On days 4 and 9, the free movement behavior was tested in an open field arena (1.5 m × 1.5 m), where each pig was recorded for 2 min in a randomized order. Tracking of the acquired videos was performed in EthoVision XT10 (Noldus Information Technology, Wageningen, Netherlands), and the total distance moved was measured [32].

When good visual function was established, the cognitive ability was assessed in a spatial plus-maze with alternating start arms functioning as a T-maze [35]. Before the cognitive testing was initiated, all pigs were habituated to new environments outside their cages once daily for three days to ensure there was no stress during the T-maze trials. As naturally reared pigs were not habituated to human handling, studies of motor activity, behavior, and cognition were restricted to only the artificially reared groups. From day 13 onwards, the pigs were tested in the maze with 10 trials per day in a randomized order for six consecutive days. A milk food reward was placed in feeding troughs in one of the two maze arms, and alternating starting positions were used, while the milk reward remained fixed in one of the maze arms. To facilitate spatial navigation, four posters with different colors and patterns were placed outside the maze as cues [36]. To reach the milk reward, the pigs had to navigate by the external maze cues using an allocentric response mechanism [35,37]. The learning criteria was defined as at least 8 correct choices out of the 10 trials [35]. All trials were recorded with EthoVision XT10 and analyzed to establish latency to choice and the distance travelled.

### 2.4. Tissue Collection

On day 19, pigs were anesthetized with an intramuscular injection of a mixture of zolazepam/tiletamin (Zoletil 50, Virbac, Kolding, Denmark), xylazine (Xysol, ScanVet, Fredensborg, Denmark), ketamine (Ketaminol, MSD Animal Health, Copenhagen, Denmark), and butorphanol (Torbugesic, ScanVet), and euthanized with intracardiac injection of sodium pentobarbital (Euthanimal, Scanvet). The brains were collected and weighed, and the hippocampus was dissected from the left cerebral hemisphere and frozen in liquid nitrogen. To estimate the cerebral water fraction, the remaining parts of the left hemisphere were weighed, dehydrated for 14 days at 50 °C and weighed again. The right cerebral hemisphere was immersed in ice-cold 4% paraformaldehyde for ex vivo structural magnetic resonance imaging (MRI).

All experimental procedures conducted during the animal experiments were approved by the Danish Animal Experiments Inspectorate (license number 2014-15-0201-00418), which is in accordance with the guidelines from Directive 2010/63/EU of the European Parliament.

### 2.5. Ex Vivo Magnetic Resonance Imaging

Ten brains from each treatment group were investigated using MRI. The paraformaldehyde-embedded right hemisphere of the brain was placed in phosphate buffer for 6 h. The MRI was conducted with a 9.4 T pre-clinical MR scanner (Agilent Technologies, Glostrup, Denmark) equipped with 4 cm diameter Millipede RF coil (Agilent Technologies) with a room temperature of 16 °C. High-resolution images were acquired with a spoiled 3D gradient echo sequence with a field-of-view (FOV) of 5 × 5 × 5 cm³ and a matrix of 192 × 192 × 192, leading to a spatial resolution of 0.26 × 0.26 × 0.26 mm^3^. Repetition time (TR) was 3.6 ms, echo time (TE) was 1.8 ms, flip angle was 30°, and 4 averages were acquired. Scan time was 8:55 minutes. Diffusion weighted imaging (dMRI) were acquired with a 2D spin echo sequence with 12 levels of diffusion sensitivity (12 b-values) and 8 diffusion directions for each level, and a b = 0 image. The diffusion directions were equally spread over the sphere and were different across the 12 b-levels (96 directions in total). FOV was 5 × 5 cm² and the matrix was 80 × 80, leading to a spatial resolution of 0.625 × 0.625 mm^2^. Slice thickness was also 0.625 mm, TR was 7.5 s and TE was 55 ms. Scan time was 16 h and 10 min, and the signal-to-noise ratio (SNR) varied between 65–85 for b = 0 and 5–10 for b = bmax, where bmax was 15,000 s/mm^2^ and the diffusion time was 40 ms.

Raw diffusion images were denoised with MP-PCA [38] and corrected for Rician noise bias [39] and Gibb’s ringing artifacts [40]. They were then fitted voxel-by-voxel to a biophysical model of diffusion in brain tissue [41,42] to obtain estimates of neurite volume fraction (water signal fraction from neurites), intra-neurite water diffusivity, and extra-neurite water diffusivity. Briefly, the biophysical model describes diffusion in brain tissue as arising from two non-exchanging main components, intra-neurite water and extra-neurite water. Each compartment is approximately characterized by Gaussian diffusion, and the net signal is obtained by convolving the underlying kernel with a fiber orientation distribution function, parameterized by a truncated Laplace series [41,42,43]. Multiple initial points for the fitting were used to ascertain the robustness of parameter estimates.

**Figure 1:**
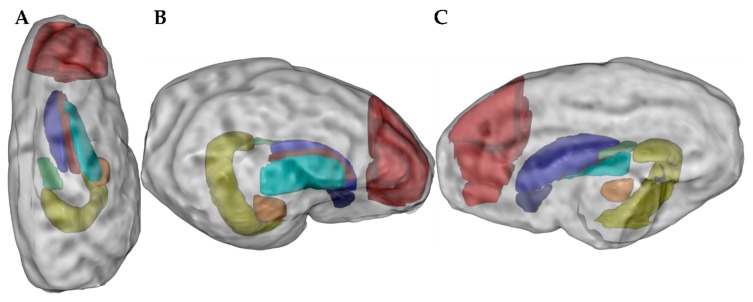
Regions of interest (ROIs) after magnetic resonance imaging presented in top view (**A**), lateral view (**B**), and medial view (**C**), respectively. Measured ROIs: nucleus accumbens (dark blue), caudate nucleus (blue), lentiform nucleus (cyan), fornix (green), hippocampus (yellow), amygdala (orange), prefrontal cortex (red), and internal capsule (burgundy).

High-resolution 3D spoiled gradient echo (SPGR) images were bias field corrected [44] and denoised [45]. For each image, a brain mask was created using fuzzy C-means followed by morphological operations and manual corrections if necessary (11 masks were manually edited). A second bias field correction was run within the mask, and an average of 15 individual images were created using an iterative framework nonlinearly, aligning the images as previously described [46]. As reference space we used a template based on in vivo MRI of the neonatal piglet available from the Pig Imaging Group at University of Illinois [47]. The template creation process was initialized by manually identifying landmarks on both the Illinois template and the individual 3D SPGR images. The result was a highly detailed ex vivo template at 200 µm isotropic resolution. Atlas labels from the Illinois template was transformed to the ex vivo template followed by manual correction. All 3D SPGR images were then nonlinearly registered to the ex vivo template using ANTS [48], and the resulting deformation fields were used to warp and resample atlas labels to dMRI space for the purpose of calculating dMRI parameters within each of the eight regions of interest (ROIs, Figure 1).

### 2.6. Sialic Acid Measurements

Gangliosides were extracted from hippocampal tissue using a modified method of Wang et al. [21], and the sediment from the processing of the ganglioside fraction was used to retrieve the protein-conjugated fraction [21]. Protein concentrations were determined in the homogenized tissues by the bicinchoninic acid assay. Quantification of sialic acid (*N*-acetylneuraminic acid (Neu5Ac) and *N*-glycolylneuraminic acid (Neu5Gc), respectively) was performed by LC–MS/MS analysis [49]. Calibration curves with concentrations of Neu5Ac (0.01–10.0 μmol/L) and Neu5Gc (0.001–1.0 μmol/L) were prepared for determining the LC–MS/MS parameters for the quantitative analyses of the protein- and ganglioside-conjugated Neu5Ac and Neu5Gc. The correlation coefficient (R^2^) of the standard curves were >0.998. Negatively single charged ions for Neu5Ac (*m*/*z* 307.7), Neu5Gc (*m*/*z* 323.7), and internal standard (*m*/*z* 310.9) were selected as parent ions and *m*/*z* 86.9, *m*/*z* 115.8, and *m*/*z* 90.0 were selected as daughter ions, respectively. The ratio of sialic acid/IS (peak area) was used to quantify the concentrations of sialic acid. The final concentrations were normalized to the total protein concentration and expressed as μg/g total protein.

### 2.7. Hippocampal Gene Expression by qPCR

For expression analysis in the hippocampus, genes of interest related to learning and memory, sialic acid and ganglioside metabolism, and neuronal development were selected together with five reference genes (Table S1). Hippocampal RNA extraction and quality analysis, cDNA synthesis, pre-amplification, and qPCR were performed [50]. Briefly, total RNA was extracted from homogenized hippocampal tissues using RNeasy Lipid Tissue Mini Kit (Qiagen, Sollentuna, Sweden). Quantity and purity of RNA were measured with NanoDrop ND-1000 UV spectrophotometer (NanoDrop Technologies, Wilmington, DE, USA) and the integrity evaluated with Agilent Bioanalyzer 2100 and RNA 6000 Nano Chips (Agilent Technologies). The cDNA was synthesized in duplicates using QuantiTect Reverse Transcription Kits (Qiagen). Pre-amplification of each cDNA was carried out using TaqMan PreAmpMasterMix (Applied Biosystems, Foster City, CA, USA) followed by exonuclease treatment (Exonuclease 1, New England biolabs, PN MO293L) [50]. Porcine-specific primers were designed using Primer3 (http://frodo.wi.mit.edu) and synthesized at Sigma-Aldrich (Brøndby, Denmark). For unvalidated primer assays, two primer pairs annealing at different sites of the mRNA transcript were designed. Transcript IDs, primer sequences, and amplicon lengths are shown in Supplementary Table S1. The qPCR of pre-amplified cDNA samples, including non-reverse transcribed as well as non-template controls, was performed using 96.96 Dynamic Array Integrated Fluidic Circuits on a BioMark thermocycler (Fluidigm, San Francisco, CA, USA) using the following cycling conditions: 2 min at 50 °C and 10 min at 95 °C, followed by 35 cycles with denaturation for 15 s at 95 °C and annealing/elongation for 1 min at 60 °C.

Data preprocessing, normalization and relative quantification were performed using GenEx5 (MultiD, Gothenburg, Sweden). The preprocessing included correction for PCR efficiency for each primer assay individually and primer amplification efficiencies between 80% and 110%, and correlations (R^2^) above 0.95 were accepted. Normalization was done to the five most consistently expressed reference genes (*ACTB, B2M, GAPDH, RPL13A, PPIA*) identified in GenEx (GeNorm and NormFinder).

### 2.8. In Vitro Oxidative Stress

To further study the effect of sialyllactose on neuronal survival, primary hippocampal neurons were isolated from Wistar rat embryos (E18; Charles River, Sulzfeld, Germany), plated at a density of 5 × 10^4^ cells/cm^2^ on poly-L-lysine-coated eight-well Lab-Tek^®^ Permanox slides (NUNC, Roskilde, Denmark) and grown for seven days [51]. Neurons were then preincubated with serially diluted SAL or lactose for 1 hour, followed by addition of freshly diluted 60 μmol/L H_2_O_2_ (Sigma-Aldrich). Cells were further cultured for 24 h, fixated with 3.7% (v/v) formaldehyde and 1% (v/v) methanol in PBS, and stained with 5 μg/mL Hoechst 33258 (Invitrogen, Taastrup, Denmark). Images of at least 500 cells/condition were recorded randomly for each group in each independent experiment (*n* = 4) using computer-assisted fluorescence microscopy [52]. Data are expressed as means of the live to total cell ratio and were normalized to unstimulated cells.

### 2.9. Statistical Analysis

Data were analyzed with the software package R (version 3.3.2, R Foundation for Statistical Computing, Vienna, Austria) unless otherwise stated, and group comparisons were performed. Gene expression data were log_2_ transformed to approach a normal distribution. Continuous data were analyzed using mixed models using birth weight as a covariate, sex, and treatment as fixed variables and litter as a random variable. Repeated measurements were analyzed with the lmer function. The proportion of pigs reaching the learning criteria per day in the T-maze was compared by logistic regression and repeated measurements. All statistical models were validated by assessment of residuals and fitted values for normality as well as variance homogeneity. Data related to the basic motor skill acquisition were handled with Mantel-Cox test in GraphPad Prism (Prism 7 for Windows, version 7.03, La Jolla, CA, USA). Probabilities lower than 0.05 were considered significant. Data are presented as raw arithmetic means and SD, unless otherwise stated. For data interpretation, the preterm intervention group PRE-SAL was compared with the preterm control group, PRE-CON. The prematurity and rearing conditions were assessed by comparing PRE-CON with TERM-CON and TERM-CON with TERM-NAT, respectively.

## 3. Results

### 3.1. Clinical Outcomes and Growth

The preterm pigs (PRE-SAL and PRE-CON) were born with an average weight of 896 ± 196 g, while the birth weight of TERM-CON was 1133 ± 166 g. The first weight recorded of TERM-NAT pigs was 1298 ± 311 g on day 3. The two preterm groups (PRE-SAL and PRE-CON) and the term control group (TERM-CON) all showed similar low growth curves with a weight gain of ~24 g/(kg*day), whereas the weight gain of the term reference pigs (TERM-NAT) was higher, i.e., ~64 g/(kg*day), (*p* < 0.001). One of the preterm litters had substantial diarrhea, and was treated with systemic antibiotics and received extended parenteral nutrition for 5 days to support the fluid and nutrition balance and ensure survival. All three artificially reared groups showed episodes of diarrhea during the study period whereas the naturally sow-reared pigs showed no clinical complications at any time.

### 3.2. Motor Acquisition, Home Cage Activity, Open Field Assessment, and Cognitive Performance

**Figure 2:**
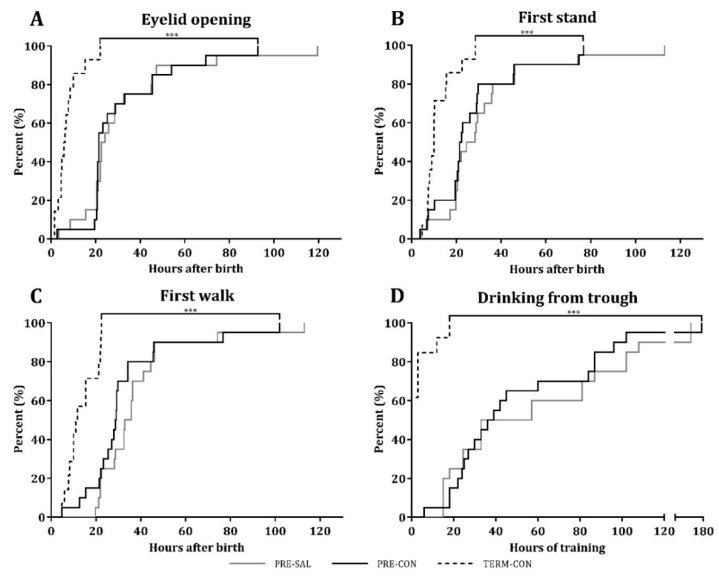
Basic motor skill acquisition. Hours before a given percentage of piglets was observed to open their eyes (**A**), stand for the first time (**B**), walk (**C**), or first voluntarily completion of a full milk bolus from an open trough (**D**). PRE-SAL (grey line), PRE-CON (black line) and TERM-CON (dotted line). *** PRE-CON and TERM-CON (*p* < 0.001).

Acquisition of basic motor skills, including first eyelid opening, first stand, first walk, as well as first voluntarily uptake of a full milk bolus from an open trough, were similar between PRE-SAL and PRE-CON (Figure 2). Indicative of the influence of prematurity per se, PRE-CON pigs used longer time than TERM-CON to acquire the abovementioned motor skills (all *p* < 0.01). Likewise, the home cage activity, as measured on days 2–9, was similar for the preterm groups PRE-SAL and PRE-CON, whereas the activity of PRE-CON pigs was lower than for TERM-CON on days 2–5 (14.6 ± 6.4 vs. 22.3 ± 9.6%, respectively, *p* < 0.001). On days 5–9, PRE-CON pigs had a slightly higher activity level than TERM-CON (24.1 ± 6.7 vs. 20.7 ± 6.2%, *p* < 0.05), most likely associated with clinical complications in the TERM-CON pigs during this period.

The open field measurements of the free movement behavior showed a general increase in the distance moved for all the groups tested from day 4 to day 9 (*p* < 0.05). Like for basic motor skills and home cage activity, the open field assessment was similar for PRE-SAL and PRE-CON pigs. Relative to the TERM-CON pigs, the PRE-CON pigs moved a shorter distance on day 4 (20.27 ± 7.76 vs. 6.74 ± 5.38 m, *p* < 0.05), while no difference was observed at day 9.

**Figure 3:**
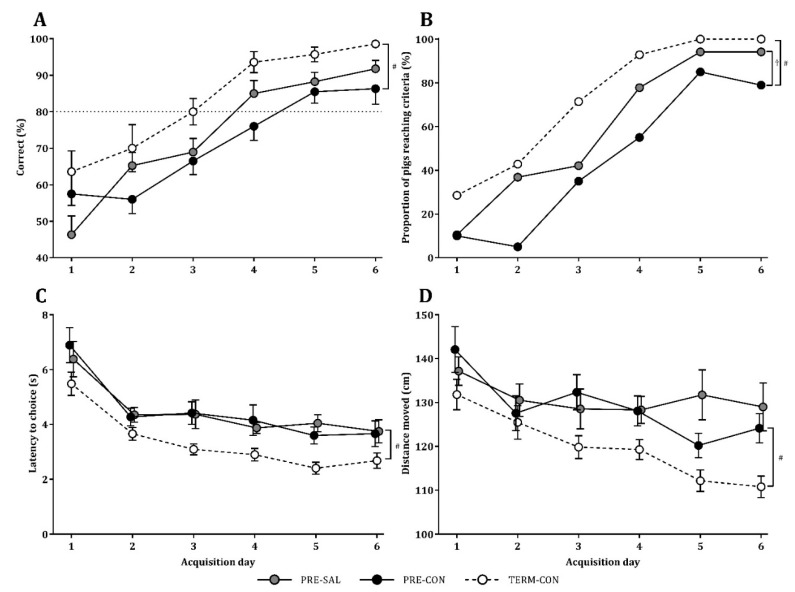
T-maze performance. Average group performances in PRE-SAL (grey), PRE-CON (black) and TERM-CON pigs (dotted line) (**A**), proportion of pigs reaching the learning criteria of 80% (**B**), latency to choice (**C**), and distance moved (**D**) during the six day acquisition period. There was a significant effect of time in all analyses (*p* < 0.001). Data are presented as mean ± SEM. ^†^ Overall difference between PRE-SAL and PRE-CON (*p* < 0.05) during the six-day test period; ^#^ Overall difference between PRE-CON and TERM-CON (*p* < 0.01).

The cognitive performance studied in the T-maze during days 13–18 showed an increase in the mean proportion of correct choices over time for all groups (*p* < 0.001). A single pig from the PRE-SAL group was excluded from the T-maze test as it only displayed off-task behavior and failed to show interest in the milk reward. The TERM-CON pigs reached the learning criteria of 80% correct choices per day at day 3, while the PRE-SAL group reached the criteria at day 4 and the PRE-CON at day 5. Over time, PRE-CON pigs showed a lower performance relative to TERM-CON pigs (*p* < 0.01, Figure 3A), while no differences were observed between PRE-SAL and PRE-CON pigs. To take into account the individual performances, the proportion of pigs that succeeded in reaching the learning criteria was determined. A higher proportion of PRE-SAL pigs reached the learning criteria relative to PRE-CON pigs (*p* < 0.05, Figure 3B), whereas the highest proportion of pigs reaching the learning criteria was met for the TERM-CON pigs (*p* < 0.01, Figure 3B). Latency to choice was shortened over time for all groups (*p* < 0.001). Across the test period, PRE-CON used longer time on decision making relative to TERM-CON (*p* < 0.01), while no difference was observed between the two preterm groups. The more rapid decision making was associated with a shorter distance moved from the starting arena to the milk reward (*p* < 0.001). Accordingly, PRE-CON pigs covered a longer distance relative to TERM-CON (*p* < 0.01), while PRE-SAL and PRE-CON were similar.

### 3.3. Brain Weights

The brain weights of PRE-SAL and PRE-CON were similar (average across groups 34.1 ± 2.4 g), whereas they were higher in the TERM-CON pigs (38.3 ± 2.0 g, *p* < 0.01). Relative to the TERM-CON pigs, TERM-NAT showed even higher brain weights of 41.5 ± 3.2 g (*p* < 0.05). The brain weight relative to body weight was similar between PRE-SAL and PRE-CON (24.2 ± 8.0 g/kg and 27.8 ± 10.0 g/kg, respectively), and between PRE-CON and TERM-CON (23.4 ± 4.4 g/kg). In contrast, TERM-CON had a higher relative brain weight than TERM-NAT (11.0 ± 4.9 g/kg, *p* < 0.001). The cerebral water fraction was also similar between PRE-SAL and PRE-CON (average across groups 82.77 ± 0.44%), while it was higher for PRE-CON relative to TERM-CON pigs (82.75 ± 0.43 vs. 81.67 ± 0.28%; *p* < 0.05). The TERM-NAT pigs also had a higher cerebral water content (82.03 ± 0.29%) relative to the TERM-CON pigs (*p* < 0.01).

### 3.4. Ex Vivo Magnetic Resonance Imaging

**Figure 4:**
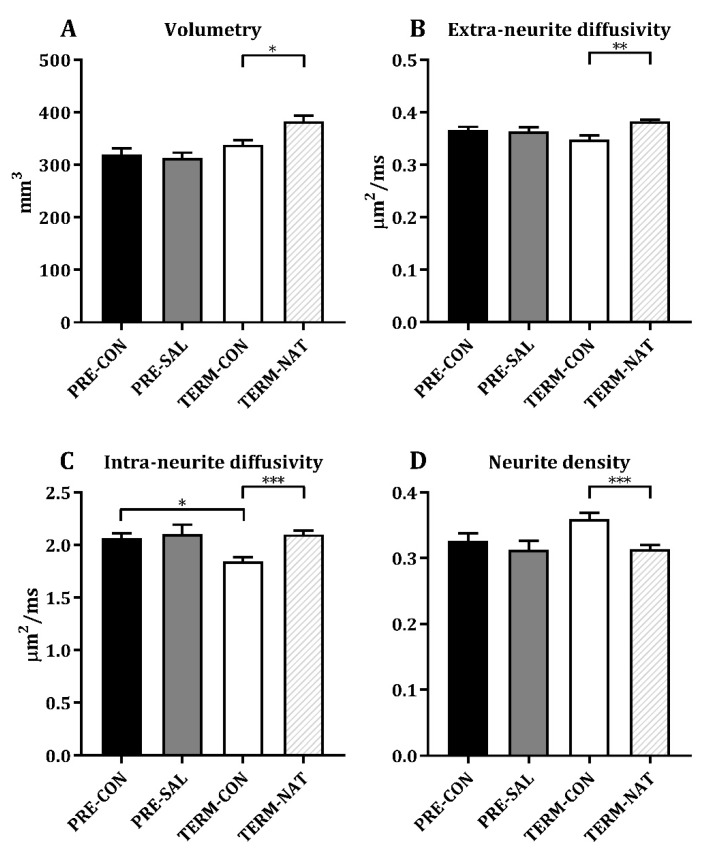
Hippocampus measurements after magnetic resonance imaging; volumes (**A**), extra-neurite diffusivity (**B**), intra-neurite diffusivity (**C**), and neurite density (**D**). Data are presented as mean ± SEM, *n* = 10 for all groups. Statistical difference * *p* < 0.05; ** *p* < 0.01; *** *p* < 0.001.

The MRI analyses were conducted on eight different ROIs as presented in Figure 4A. The results were similar between PRE-SAL and PRE-CON for both volumetric and diffusion weighted measures. Results from all measured ROIs are listed in Table 1 and data from the hippocampus are shown in Figure 4. No difference was observed on the volumetric measures for all ROIs between PRE-CON and TERM-CON. The PRE-CON pigs showed a higher extra-neurite diffusivity in caudate nucleus, lentiform nucleus, prefrontal cortex, as well as the internal capsule (all *p* < 0.05, Table 1). The intra-neurite diffusivity was higher for PRE-CON relative to TERM-CON in nucleus accumbens, caudate nucleus, hippocampus, amygdala, prefrontal cortex, and in the internal capsule (all *p* < 0.05, Table 1 and Figure 4D). Furthermore, PRE-CON showed a lower neurite density in nucleus accumbens, caudate nucleus, prefrontal cortex and in the internal capsule relative to TERM-CON (Table 1). Relative to TERM-CON, the TERM-NAT pigs had larger hippocampus and prefrontal cortex (*p* < 0.05 and *p* < 0.001, respectively, Table 1 and Figure 4B). TERM-CON showed lower extra-neurite diffusivity and lower intra-neurite diffusivity for all measured ROIs apart from fornix, relative to TERM-NAT (Table 1 and Figure 4C,D). Furthermore, TERM-CON had higher neurite densities compared to TERM-NAT in nucleus accumbens, caudate nucleus, lentiform nucleus, hippocampus, amygdala, and in the prefrontal cortex (Table 1 and Figure 4E).

### 3.5. Sialic Acid Levels in Hippocampal Tissue

**Figure 5:**
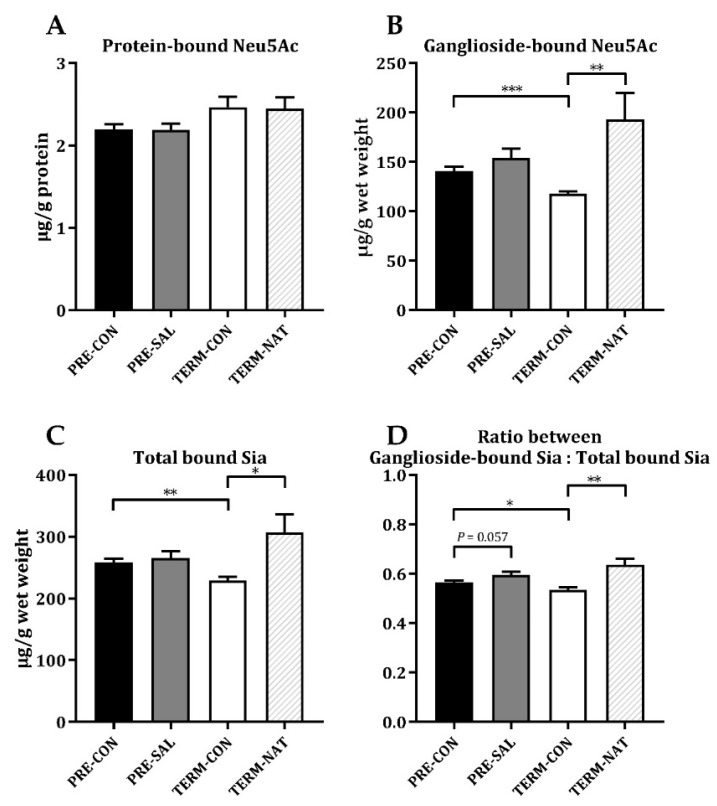
Sialic acid levels in hippocampal tissue. Protein-bound Neu5Ac (**A**), ganglioside-bound Neu5Ac (**B**), total levels of bound sialic acid (**C**), and ratio between the ganglioside-bound and total levels of bound sialic acids (**D**). Abbreviations: Neu5Ac; N-acetylneuraminic acid, Sia; sialic acid. Data are means ± SEM. Statistical difference * *p* < 0.05; ** *p* < 0.01; *** *p* < 0.001.

Whereas fortification with sialyllactose did not increase the sialic acid levels in hippocampus of PRE-SAL (Figure 5A–C), there was a tendency toward a higher ratio of ganglioside-bound sialic acid relative to the total bound fraction of sialic acids relative to PRE-CON (*p* = 0.057, Figure 5D). For the protein-bound fraction of Neu5Gc, PRE-SAL had decreased levels relative to PRE-CON (*p* < 0.05, Table 2), while similar levels were observed in the ganglioside-bound fraction. Likewise, the total levels of both protein-bound and ganglioside-bound sialic acid did not differ among the preterm groups (Table 2). Relative to TERM-CON, no differences were observed for PRE-CON for the protein-bound fractions of sialic acid. The PRE-CON had higher levels of ganglioside-bound Neu5Ac, as well as the total level of ganglioside-bound sialic acids (both *p* < 0.001, Figure 5B and Table 2) and the total level of bound sialic acids was higher in PRE-CON relative to TERM-CON (*p* < 0.01, Figure 5C). The PRE-CON group had a higher ratio between ganglioside-bound and the total bound fraction of sialic acid relative to TERM-CON (*p* < 0.05 Figure 5D). For term pigs, TERM-CON showed decreased levels of the protein-bound fractions of Neu5Gc relative to TERM-NAT (*p* < 0.05, Table 2), but similar levels of the protein-bound fraction of Neu5Ac and the total protein-bound level of sialic acid. For all ganglioside-bound fractions, TERM-CON had decreased levels relative to TERM-NAT (all *p* < 0.01, Figure 5B and Table 2).

### 3.6. Hippocampal Gene Expression

**Figure 6:**
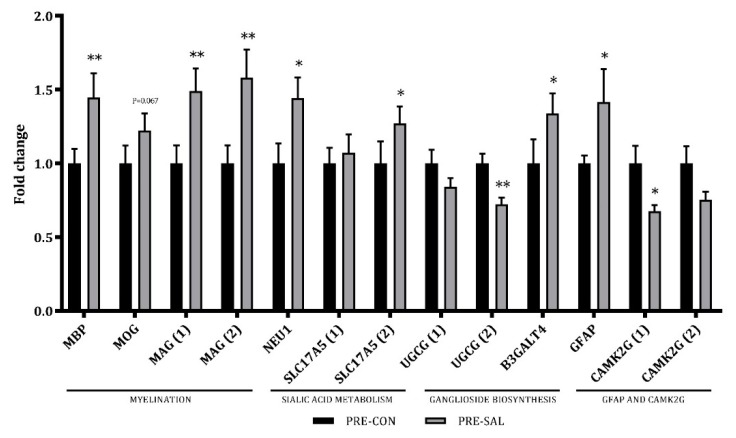
Gene expression in hippocampal tissue. Relative mRNA expression levels of genes related to myelination, sialic acid metabolism, and ganglioside biosynthesis. Numbers in brackets indicate primer number if several primers for same gene were tested. Data are means ± SEM. Statistical difference * *p* < 0.05; ** *p* < 0.01.

In hippocampus, the expression of genes related to learning and memory, sialic acid and ganglioside metabolism, and neuronal development were assessed. Whereas sialyllactose enrichment did not modify the expression of pre-selected genes involved in memory formation and learning processes (*BDNF*, *NCAM1*, *NCAM2*, *GAP43*, and others), it changed the expression of eight genes, including those involved in myelination and sialic acid metabolism (Figure 6). The expression level of mRNA encoding for glial fibrillary acidic protein gene (*GFAP*), the myelination-responsible genes, myelin-associated glycoprotein (*MAG*), and myelin basic protein (*MBP*) were upregulated 1.42-fold (*p* < 0.05), 1.5-fold (*p* < 0.01), and 1.45-fold (*p* < 0.01) in the hippocampus of PRE-SAL versus PRE-CON pigs, respectively (Figure 6). The expression of myelin oligodendrocyte glycoprotein (*MOG*) also tended to be modestly upregulated in the PRE-SAL pigs (1.22-fold increase, *p* = 0.067). In addition, the genes *NEU1*, encoding neuraminidase 1 protein, and *SLC17A5*, encoding Sialin, were upregulated 1.45- and 1.27-fold, respectively (*p* < 0.05). Moreover, genes involved in ganglioside biosynthesis changed expression for PRE-SAL, i.e., *UGCG*, encoding UDP-glucose ceramide glucosyltransferase, was downregulated by 39% (*p* < 0.01), and *B3GALT4*, encoding beta-1,3-galactosyltransferase 4, was upregulated (1.34-fold increase, *p* < 0.05). Finally, calcium/calmodulin dependent protein kinase II gamma (*CAMK2G*) expression in the hippocampus was downregulated by 47% (*p* < 0.05; Figure 6).

Differences in gene expression according to the gestational age was compared and three genes encoding the light, medium, and heavy chain of three neurofilament proteins (*NEFL*, *NEFM*, and *NEFH*) were all downregulated in TERM-CON relative to PRE-CON pigs with fold changes of −6.3, −2.7, and −3.4, respectively (all *p* < 0.001). As observed for PRE-SAL, the TERM-CON group also showed an upregulation of GFAP (1.23-fold increase, *p* < 0.001) and NEU1 (1.22-fold increase, *p* < 0.05), relative to PRE-CON. Remaining genes differentially regulated between the PRE-CON and TERM-CON pigs are listed in Table 3.

The TERM-NAT pigs with natural rearing conditions, showed an upregulation of genes encoding proteins involved in myelination (*MBP, PLP1, MOG*, and *MAG*) relative to the artificially reared TERM-CON pigs with fold-changes of 8.3, 3.8, 3.8, and 2.3, respectively (all *p* < 0.001). Furthermore, as observed with PRE-SAL, *GFAP* and *SLC17A5* were upregulated in TERM-NAT relative to TERM-CON. The remaining genes differentially regulated between the TERM-NAT and TERM-CON pigs are shown in Table 4.

### 3.7. SAL and Lactose Promote Neuronal Survival In Vitro

**Figure 7:**
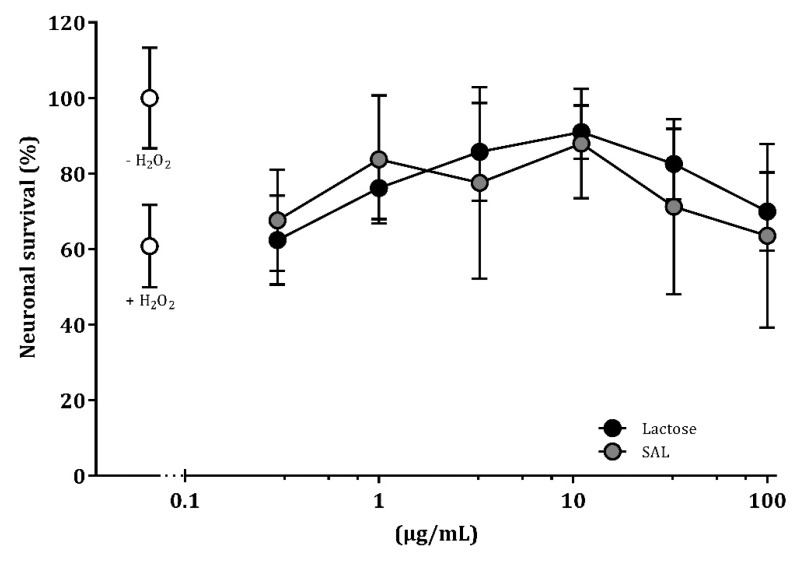
Dose–response effect of sialyllactose and lactose on neuronal survival. Differentiated hippocampal neurons were incubated with serially diluted oligosaccharide-enriched whey protein concentrate with sialyllactose (SAL) or lactose and treated with H_2_O_2_ for 24 h. Treatment with H_2_O_2_ alone resulted in 40% neuronal death compared to untreated control neurons (set to 100% survival, *p* < 0.001). SAL and lactose reduced cell death from H_2_O_2_ treatment at concentrations of 1–11 μg/mL and 3.3–11 μg/mL, respectively (*p* < 0.05). No difference was observed between SAL and lactose. Data are means ± SEM (*n* = 4).

Cell death was induced by challenging hippocampal neurons with H_2_O_2_ and resulted in 40% neuronal death compare to unstimulated control neurons. Treatment with SAL or lactose reduced cell death and thereby promoted neuronal survival at a concentration range of 1–11 μg/mL and 3.3–11 μg/mL, respectively, compared to H_2_O_2_-challenged control neurons (*p* < 0.05; Figure 7).

## 4. Discussion

We investigated the neurodevelopmental effects of dietary supplementation with a sialic acid-enriched whey ingredient in preterm pigs, and found that more SAL-supplemented preterm pigs reached the learning criteria in a T-maze relative to control preterm pigs, approaching the cognitive level of corresponding term pigs. Further, SAL supplementation resulted in the regulation of genes related to sialic acid metabolism, myelination, and ganglioside biosynthesis. Whereas growth and structural hippocampal indices were improved in naturally reared term pigs, artificial rearing reduced clinical indices and growth, but maintained cognitive performance.

The artificially reared pigs were fed a bovine milk-based diet supplemented with either lactose or an oligosaccharide-enriched whey with sialyllactose, including both 3’SL and 6’SL. The level of sialic acid was four times higher in the SAL vs. CON diet (433 and 120 mg/L, respectively). Sialyllactose is a predominant sialylated oligosaccharide in human milk. The total levels of 3’- and 6’SL in human milk start at 690–880 mg/L at day 3–5 after parturition, and are reduced to 430 mg/L at day 60 [53,54]. The dose used in the present study corresponds to levels found in mature human milk. In comparison, sialic acid concentrations in infant formulas are <25% of that in mature human milk [17]. We formulated the diet with levels substantially higher than what is found in infant formulas to secure biological relevance of the study without the risk of adverse effects in the sensitive preterm pigs, and the gastrointestinal effects have been evaluated recently [34]. Essential nutrients, such as sialic acid, have been shown to enhance brain development, cognition, and memory in animals [25,55]. Likewise, adequate nutrient intake in preterm infants revealed a higher intelligence quotient at 7–8 years of age compared with basic formula feeding [56]. The period around birth is particularly important as rapid brain growth requires precursors to support neurodevelopment, and nutrient deficiencies can have severe consequences for cognitive development [57]. Therefore, cognitive function is an appropriate endpoint to document the effects of dietary compounds like sialyllactose in the neonatal period. Among the oligosaccharides in the whey ingredient, we speculate that the effects observed on cognition and brain gene expression was mainly associated with sialyllactose, since sialic acid has been identified as an essential nutrient for brain development and cognition.

The cognitive abilities were assessed in a spatial T-maze, and all groups showed an increased mean proportion of correct choices over time, indicating an allocentric response strategy of the pigs [37]. The average daily performance is sensitive to individual pigs with either very low or high performance. Therefore, we also compared the proportion of animals that reached the learning criterion. A higher proportion of PRE-SAL pigs reached the learning criteria compared with PRE-CON. Cognitive ability in artificially reared term pigs has previously been estimated using the T-maze [35,58], and sialic acid has improved spatial learning in an 8-armed radial maze in term pigs [29] and adult rats [25]. Others do not find sialyllactose to improve recognition memory in young pigs [59]. We found that preterm pigs (PRE-CON) were inferior to term-born reference pigs (TERM-CON) in the spatial T-maze. Whereas previous data show delayed motor and behavioral skills in preterm pigs [32], the current data are the first to document impaired cognitive ability in preterm versus term pigs by performance in a T-maze. It is plausible that the levels of comorbidity may have influenced the results. This is in line with observations in preterm versus term infants [4,5].

The other indicators of brain development, i.e., cerebral water content, basic motor skills, home cage activity, and open field assessment, were similar for PRE-SAL and PRE-CON. Any possible effect of sialyllactose on these parameters in the doses given may require longer exposure for effects to appear. The PRE-CON was generally inferior to TERM-CON which is in line with previous observations [32]. Moreover, the higher water content in the preterm pigs corresponds to observations in pigs and humans [60,61]. While there were no major clinical complications during the first days after birth, later episodes of diarrhea appeared among the artificially reared pigs. One litter of preterm pigs, in particular, was in need of additional antibiotic treatment. Despite the immunization with maternal plasma, the cesarean-derived pigs showed increased gut sensitivity, which reduced their cognitive performance. On the contrary, the naturally reared TERM-NAT pigs had no clinical complications.

SAL supplementation did not affect the MRI analyses. Previously, changes in diffusivity in corpus callosum have been observed after comparable doses of SAL administration [62]. Here, MRI scans were only performed on a single hemisphere from each animal; hence, data from corpus callosum could not be obtained. No volumetric differences were observed between PRE-CON and TERM-CON. Reduced volumes in several brain regions, including hippocampus, has been observed in preterm infants [63], and their brain diffusivity is associated with age [64]. The water content decreases during maturation of the human preterm brain [61]. Changes in water content as well as the development of white and grey matter structures is thought to affect the tissue microstructure, diffusivity, and anisotropic diffusion [65,66], and the diffusivity of the preterm brain is associated with age [64]. Preterm infants show lower anisotropy in several white matter structures [67,68], decreased fractional anisotropy, and increased radial diffusivity in specific white matter regions has been related to cognitive and motor outcomes at 2-year corrected age [69]. In this study, we observed increased extra-neurite diffusivity for PRE-CON relative to TERM-CON in caudate nucleus, lentiform nucleus, the prefrontal cortex, as well as the internal capsule. Furthermore, the preterm animals had, in general, a higher intra-neurite diffusivity across many of the measured ROIs, but also lower neurite densities relative to the term controls.

Within the central nervous system, sialic acid is mainly present in the gangliosides, particularly in the gray matter [70]. An increase in brain gangliosides is associated with both growth and maturation [71]. In the current study, no changes in the ganglioside-bound fractions of sialic acid in hippocampus were observed after supplementation with SAL. However, PRE-SAL tended to have a higher ratio of ganglioside-bound sialic acids relative to the total fraction of bound sialic acids compared to PRE-CON. The fraction of free sialic acid in hippocampus was not measured. A previous study documented changes in the ratio between free and bound sialic acid in hippocampus [62]. The PRE-CON piglets had more ganglioside-bound sialic acid than TERM-CON although both groups were fed the same diet. On the contrary, TERM-NAT pigs fed by the sow had significantly higher levels. This reflects the higher sialic acid content in sows milk compared with bovine milk [72]. Supplementation with SAL increased expression of genes involved in myelination processes and sialic acid metabolism. The genes involved in myelination, as well as GFAP and SLC17A5, were also increased in TERM-NAT pigs.

It is not known if the effects would persist beyond the 19 days. In humans, it has been reported that some preterm infants with cognitive deficits show progress over time and develop cognitive skills comparable with term peers [73]. The preterm pigs were chosen as a model due to their resemblance in brain structure and function to preterm infants [74,75,76], and similar to infants, we found that preterm pigs were able to perform the same task as the term-born reference pigs, albeit at an inferior level. It remains a question whether the preterm pigs are able to catch up with the term reference pigs on their cognitive ability at a later time point than the current study period of 19 days. Also, the nature of the sialic acid blend, per se, may influence the early life neurodevelopment since, e.g., sialyllactose in rodents have shown shorter bowel retention time as well as lower digestion rates than sialylated glycoproteins [77].

## 5. Conclusions

To our best knowledge, this is the first study to investigate the early postnatal effects on brain development in a preterm pig model after supplementation with a milk ingredient rich in sialyllactose. The oligosaccharide-enriched whey with sialyllactose elicited positive functional brain effects and increased the expression of myelination genes in the hippocampus at 3 weeks of age. Sialyllactose may therefore be an important supplement to infant formula to stimulate preterm neonatal brain development. Further studies are required to establish the specific sialyllactose effects after administration as well as the optimal dose and timing of supplementation.

## Figures and Tables

**Table 1:** Magnetic resonance imaging for the ROIs not shown in Figure 4.

**Volumetry (mm^3^)**	**PRE-CON**		**PRE-SAL**		**TERM-CON**		**TERM-NAT**	
Nucleus accumbens	36.8 ± 4.9		38.8 ± 6.1		41.2 ± 2.8		43.5 ± 5.0	
Caudate nucleus	189 ± 14		196 ± 17		213 ± 14		231 ± 16	
Lentiform nucleus	203 ± 21		196 ± 22		217 ± 13		234 ± 15	
Fornix	16.8 ± 3.0		16.7 ± 2.6		19.3 ± 2.6		19.2 ± 2.8	
Amygdala	53.8 ± 7.6		54.8 ± 7.8		56.9 ± 6.4		64.6 ± 7.0	
Prefrontal cortex	1050 ± 104		1043 ± 92		1109 ± 78		1280 ± 72	^∆∆∆^
Internal capsule	92.8 ± 11.5		95.2 ± 10.5		100.8 ± 6.4		110.7 ± 5.9	
**Extra-neurite diffusivity (µm^2^/ms)**								
Nucleus accumbens	0.352 ± 0.026		0.353 ± 0.025		0.328 ± 0.020		0.356 ± 0.015	^∆∆^
Caudate nucleus	0.358 ± 0.018		0.357 ± 0.026		0.331 ± 0.013	^§^	0.369 ± 0.020	^∆∆∆^
Lentiform nucleus	0.362 ± 0.032		0.358 ± 0.028		0.326 ± 0.009	^§^	0.372 ± 0.013	^∆∆∆^
Fornix	0.441 ± 0.148		0.432 ± 0.110		0.658 ± 0.503		0.409 ± 0.109	
Amygdala	0.373 ± 0.043		0.362 ± 0.038		0.346 ± 0.014		0.381 ± 0.014	^∆∆∆^
Prefrontal cortex	0.363 ± 0.026		0.366 ± 0.023		0.340 ± 0.005	^§^	0.367 ± 0.013	^∆∆∆^
Internal capsule	0.306 ± 0.043		0.304 ± 0.028		0.274 ± 0.013	^§^	0.313 ± 0.021	^∆∆∆^
**Intra-neurite diffusivity (µm^2^/ms)**								
Nucleus accumbens	2.08 ± 0.36		2.08 ± 0.47		1.63 ± 0.23	^§^	2.06 ± 0.22	^∆∆∆^
Caudate nucleus	1.90 ± 0.13		1.85 ± 0.31		1.58 ± 0.13	^§^	1.82 ± 0.19	^∆∆∆^
Lentiform nucleus	1.82 ± 0.23		1.77 ± 0.26		1.52 ± 0.10		1.74 ± 0.12	^∆∆∆^
Fornix	2.00 ± 0.27		1.92 ± 0.17		1.93 ± 0.40		2.10 ± 0.47	
Amygdala	2.25 ± 0.33		2.22 ± 0.43		1.87 ± 0.19	^§^	2.28 ± 0.23	^∆∆∆^
Prefrontal cortex	2.57 ± 0.18		2.55 ± 0.39		2.16 ± 0.09	^§§§^	2.44 ± 0.13	^∆∆∆^
Internal capsule	1.39 ± 0.09		1.36 ± 0.15		1.20 ± 0.07	^§^	1.33 ± 0.09	^∆∆∆^
**Neurite density**								
Nucleus accumbens	0.273 ± 0.036		0.279 ± 0.044		0.321 ± 0.022	^§§§^	0.286 ± 0.041	^∆^
Caudate nucleus	0.293 ± 0.029		0.286 ± 0.038		0.343 ± 0.018	^§§^	0.297 ± 0.030	^∆∆∆^
Lentiform nucleus	0.291 ± 0.036		0.265 ± 0.026		0.331 ± 0.029		0.296 ± 0.025	^∆∆^
Fornix	0.641 ± 0.050		0.640 ± 0.065		0.601 ± 0.119		0.651 ± 0.055	
Amygdala	0.293 ± 0.053		0.262 ± 0.051		0.310 ± 0.024		0.282 ± 0.026	^∆^
Prefrontal cortex	0.287 ± 0.038		0.350 ± 0.232		0.336 ± 0.020	^§^	0.295 ± 0.015	^∆∆∆^
Internal capsule	0.499 ± 0.035		0.486 ± 0.033		0.549 ± 0.038	^§^	0.518 ± 0.049	

Difference between PRE-CON and TERM-CON, ^§^ *p* < 0.05; ^§§^ *p* < 0.01; ^§§§^ *p* < 0.001. Difference between TERM-CON and TERM-NAT, ^∆^ *p* < 0.05; ^∆∆^ *p* < 0.01; ^∆∆∆^ *p* < 0.001. Values are presented as mean ± SD.

**Table 2:** Sialic acid levels in hippocampal tissue. Values for protein-bound fractions are presented as µg/g protein whereas ganglioside-bound fractions are presented as µg/g wet weight tissue.

	PRE-CON		PRE-SAL		TERM-CON		TERM-NAT	
Protein-bound Neu5Gc	0.099 ± 0.018	^€^	0.085 ± 0.023		0.129 ± 0.033		0.162 ± 0.019	^∆^
Total protein-bound sialic acid	2.33 ± 0.28		2.25 ± 0.35		2.59 ± 0.48		2.61 ± 0.45	
Ganglioside-bound Neu5Gc	5.63 ± 1.69		5.64 ± 2.42		4.23 ± 0.68		8.18 ± 4.34	^∆^
Total ganglioside-bound sialic acid	146 ± 19		159 ± 43		122 ± 9	^§§§^	201 ± 90	^∆∆^

Difference between PRE-CON and PRE-SAL, ^€^ *p* < 0.05. Difference between PRE-CON and TERM-CON, ^§§§^ *p* < 0.001. Difference between TERM-CON and TERM-NAT, ^∆^ *p* < 0.05; ^∆∆^ *p* < 0.01. Abbreviations: Neu5Gc; N-glycolylneuraminic acid. Values are presented as mean ± SD.

**Table 3:** Relative mRNA expression levels of selected genes for TERM-CON. Values are scaled to PRE-CON set to 1. Numbers in brackets indicate primer number if several primers for the same gene were tested.

Gene Symbol	Gene Name	Fold Change	*p*-Value
***NTRK2 (2)***	Neurotrophic receptor tyrosine kinase 2	−1.594	<0.05
***DLG4 (1)***	Discs, large homolog 4	1.326	<0.05
***NCAM1***	Neural cell adhesion molecule 1	1.135	<0.05

**Table 4:** Relative mRNA expression levels of selected genes for TERM-NAT. Values are scaled to TERM-CON set to 1. Numbers in brackets indicate primer number if several primers for same gene were tested.

Gene Symbol	Gene Name	Fold Change	*p*-Value
*NCS1 (1)*	Neuronal calcium sensor 1	1.871	<0.01
*NSC1 (2)*		3.063	<0.001
*ST3GAL3*	ST3 beta-galactoside alpha-2,3-sialyltransferase 2	2.625	<0.001
*CAMK2A (1)*	Calcium/calmodulin-dependent protein kinase II alpha	2.055	<0.001
*CAMK2A (2)*		2.441	<0.001
*DCX*	Doublecortin	2.469	<0.001
*NEFH*	Neurofilament heavy chain	2.039	<0.001
*GRIA4 (1)*	Glutamate receptor, ionotropic, AMPA 4	1.944	<0.001
*CREB1 (1)*	cAMP-responsive element binding protein 1	1.678	<0.001
*CREB1 (2)*		1.788	<0.001
*VEGFA*	Vascular endothelial growth factor A	1.752	<0.001
*SLC2A3/GLUT3*	Solute carrier family 2 member 3	1.643	<0.05
*FGF2 (2)*	Fibroblast growth factor 2	1.636	<0.001
*NCAM2 (1)*	Neural cell adhesion molecule 2	1.619	<0.001
*NCAM2 (2)*		1.631	<0.001
*NTRK2 (1)*	Neurotrophic receptor tyrosine kinase 2	1.574	<0.001
*NTRK2 (2)*		1.476	<0.001
*SYNPO*	Synaptopodin	1.494	<0.01
*BDNF (1)*	Brain-derived neurotrophic factor	−1.410	<0.05
*B4GALNT1*	Beta-1,4-*N*-acetyl-galactosaminyl transferase 1	1.381	<0.01
*OCLN*	Occludin	1.368	<0.01
*DLG4 (2)*	Discs, large homolog 4	1.352	<0.05
*NCAM1*	Neural cell adhesion molecule 1	1.218	<0.05

## Supplementary Materials

The following are available online at https://www.mdpi.com/2072-6643/11/6/1335/s1, Table S1: Transcript IDs, primer sequences and amplicon lengths of qPCR analyzed genes in hippocampal tissue.
