# Supplementary material for: Bovine Milk Oligosaccharides with Sialyllactose Improves Cognition in Preterm Pigs
Source: Nutrients. 2019 Jun 14;11(6):1335. doi: 10.3390/nu11061335 (PMC6628371; doi:10.3390/nu11061335)
Supplement: Supplementary file 1 [file nutrients-11-01335-s001.pdf]

nutrients MDPI

Supplementary Materials

Supplementary table 1. Transcript IDs, primer sequences and amplicon lengths of qPCR analyzed genes in hippocampal tissue.

Gene symbol	Gene name	Sequence (5' to 3')	Sequence (5' to 3')	Amplicon length
ACTB	Actin beta	F: CTACGTCGCCCTGGACTTC	R: GCAGCTCGTAGCTCTTCTCC	76
B3GALT4	UDP-Gal:BetaGlcNAc Beta 1,3-Galactosyltransferase, polypeptide 4	F: CTGAACTGGGCTGACAAACA	R: CTGGGACATTGACAAACACG	79
B4GALNT1	Beta-1,4-N-Acetyl-Galactosaminyl Transferase 1	F: CAGGGAGCCCAGTACAACAT	R: GCCGATTGTAACGAAGGAAG	70
B2M	Beta-2-Microglobulin	F: TGAAGCACGTGACTCTCGAT	R: CTCTGTGATGCCGGTTAGTG	70
BDNF (1)	Brain Derived Neurotrophic Factor	F: CCTTTGGAGCCTCCTCTTCT	R: CCTCATGGACATGTTTGCAG	75
BDNF (2)	Brain Derived Neurotrophic Factor	F: TTGAACACGTGATCGAGGAG	R: TCCGCGTCCTTATTGTTTTC	76
CAMK2A (1)	Calcium/Calmodulin-Dependent Protein Kinase II Alpha	F: ACAGACAGGAGACCGTGGAC	R: AGCATGGTGGTGAGGATAGC	79
CAMK2A (2)	Calcium/Calmodulin-Dependent Protein Kinase II Alpha	F: AGCTATCCTCACCACCATGC	R: CGCCGTCATTCTTCTTGTTT	77
CAMK2B (1)	Calcium/Calmodulin-Dependent Protein Kinase II Beta	F: CCCAGACGAATAGCACCAAA	R: GGGTTATGAATGACGGTGGT	100
CAMK2B (2)	Calcium/Calmodulin-Dependent Protein Kinase II Beta	F: GGCAATCTGGTTGAAGGAAT	R: GTTCAGGATGGTTGTGTGGA	93
CAMK2G (1)	Calcium/Calmodulin-Dependent Protein Kinase II Gamma	F: TCACCTCCTTTGAGCCTGAG	R: GGGCTTGCTGTTCTTGGATA	98
CAMK2G (2)	Calcium/Calmodulin-Dependent Protein Kinase II Gamma	F: GGACTTTGAGGCCTACACGA	R: CTTCCACCAGGTTACCAAGG	80
CREB1 (1)	cAMP Responsive Element Binding Protein 1	F: GGCCTGCAGACATTAACCAT	R: TCTGCTGTCCATCAGTGGTC	91
CREB1 (2)	cAMP Responsive Element Binding Protein 1	F: ACTGTAACGGTGCCAACTCC	R:ATTGCTCCTCCCTGGGTAAT	74
DCX	Doublecortin	F: CCTCAGGGAGTGCGTTACAT	R: ATAGCTTTCCCCTTCCTCCA	84
DLG4 (1)	Discs, Large homolog 4	F: AGAAGGACATCCAGGCACAC	R: ACTGGACGCTGGTTCCATAC	75
DLG4 (2)	Discs, Large homolog 4	F: TCCTATCGCCATCTTCATCC	R: GCTCCTCTGTGATCCGCTTA	74
FGF2 (1)	Fibroblast Growth Factor 2	F: CGGTCGAGGAAATACTCCAG	R: TCCTGTTTTGGGTCCAAGTT	75
FGF2 (2)	Fibroblast Growth Factor 2	F: AAGCAGAAGAGAGAGGGGTTG	R: GCCAGTAATCTTCCATCTTCCTT	85
GAP43	Growth Associated Protein 43	F: AAAGCTTCCACTGACAACTCG	R: GGCTTGTTTAGGCTCCTCCT	72

GAPDH	Glyceraldehyde-3-phosphate dehydrogenase	F: ACCCAGAAGACTGTGGATGG	R: AAGCAGGGATGATGTTCTGG	79
GFAP	Glial fibrillary acidic protein	F: ACATCGAGATCGCCACCTAC	R: GCAGATTGGAGAAGGTCTGC	84
GRIA1	Glutamate Ionotropic Receptor AMPA Type Subunit 1	F: AGGGCAAATACGCCTACCTT	R: CCACCTTCATGGTGTCACAG	81
GRIA2	Glutamate Ionotropic Receptor AMPA Type Subunit 2	F: CCTCGGTGTTTGTGAGGACT	R: GTGGACTCCAGCAAGTAGGC	85
GRIA3	Glutamate Ionotropic Receptor AMPA Type Subunit 3	F: CTGCTGGAGTCAACCATGAA	R: GGAATCCAGATTTCCACCAA	81
GRIA4 (1)	Glutamate Ionotropic Receptor AMPA Type Subunit 4	F: TAGCTCGTGTCCGAAAATCC	R: CATCGTGTCACATGGCTTTC	95
GRIA4 (2)	Glutamate Ionotropic Receptor AMPA Type Subunit 4	F: GGGCAGAAGCAAAGAGAATG	R: TTTTCACCCACACTCCCAGT	88
GRIN2A (1)	Glutamate Receptor, Ionotropic, N-Methyl D-Aspartate 2A	F: ACGGGCCTTCTTTTACGATT	R: GCTGGTGGTCCCTTTAGGAT	98
GRIN2A (2)	Glutamate Receptor, Ionotropic, N-Methyl D-Aspartate 2A	F: TGATGCTGCTCATTGTCTCC	R: TGCTTTCCCTTTGGCTAAGT	92
GRIN2B	Glutamate Receptor, Ionotropic, N-Methyl D-Aspartate 2B	F: GTCACGCAGAACCCTTTCAT	R: GTGGGCTGCCTGAAGAAGTA	86
FAIM2	Fas apoptotic inhibitory molecule 2	F: TACAACACCACATCCGTGCT	R: GTCGAACTTGGTCTGGAAGC	90
MAG (1)	Myelin Associated Glycoprotein	F: TCTTGTGCTCCACACAGAGC	R: ACAGTGGCCAGAATCTGCTT	73
MAG (2)	Myelin Associated Glycoprotein	F: GTGGAGCTCAGCGTCATGTA	R: CTGTGTGGAGCACAAGATGG	99
MAP2	Microtubuli-Associated Protein 2	F: AGCCTAAAGGGGGACAGGTA	R: GGCGGATGTTCTTCAGAGAG	90
MBP	Myelin Basic Protein	F: TGACTACAAACCGGCTCACA	R: TCCCAGCTTGAAGATTTTGG	79
MOG	Myelin oligodendrocyte glycoprotein	F: CGAGCAGAGATCGAGAACCT	R: AACTGGCACGATCACAAACA	93
NCAM1	Neural Cell Adhesion Molecule 1	F: GTGCAGTTTGATGAGCCAGA	R: ACCACTTGGAGTGCCAGACT	100
NCAM2 (1)	Neural Cell Adhesion Molecule 2	F: AGGGTTGCAGCTGTAAATGG	R: CACTGCTAGGCTGTCCATGA	115
NCAM2 (2)	Neural Cell Adhesion Molecule 2	F: AGCCAAGGTTTCTTTCAGCA	R: CCACCTCTTTGACGTCCACT	80
NCS1 (1)	Neuronal Calcium Sensor 1	F: TGTGGATGCCATTTACCAGA	R: GTCCATCATGGCAAAGATCC	100
NCS1 (2)	Neuronal Calcium Sensor 1	F: TAACGACGGCTACATCACCA	R: GTTGCCCACCATCTGGTAAA	70
NEFL	Neurofilament light chain	F: AGAACGCCGACATTAGTGCT	R : TGCCATTTCACTCTTTGTGG	80
NEFM	Neurofilament medium chain	F: AGGAGAAGCCACAGCAGAAG	R: TTCCTTCAAACCTCCCTCCT	71
NEFH	Neurofilament heavy chain	F: CAGGACCTGCTCAATGTCAA	R: CGAAGCCAATTCGACACTCT	91
NEU1	Neuraminidase 1	F: CCGGAACCAGAACAACTACC	R: GGGGTCAAAGGTCACATCAC	97
NTRK2 (1)	Neurotrophic Receptor Tyrosine Kinase 2	F: TTGTGTGGCAGAAAATCTCG	R: GGTCTGAGGTTGGAGATTCG	101

NTRK2 (2)	Neurotrophic Receptor Tyrosine Kinase 2	F: GGGGCAATTTGAATGAGTC	R: CGTGGTACTCCGTGTGATTG	70
NTF3	Neurotrophin 3	F: AGACTCGCTCAATTCCCTGA	R: CTGAAGGTCCACCATCTGCT	85
OCLN	Occludin	F:GACGAGCTGGAGGAAGACTG	R: GTACTCCTGCAGGCCACTGT	102
PLP1	Proteolipid Protein 1	F: TTGCCAGTCTATTGCCTTCC	R: GAAAGCATTCCATGGGAGAA	97
PPIA	Peptidylprolyl isomerase A (cyclophilin A)	F: CAAGACTGAGTGGTTGGATGG	R: TGTCCACAGTCAGCAATGGT	138
RPL13A	Ribosomal protein L13a	F: ATTGTGGCCAAGCAGGTACT	R: AATTGCCAGAAATGTTGATGC	76
SLC17A5 (1)	Solute Carrier Family 17 member 5	F: CTATTCCCTGGCTGTTGCAT	R: CAATGTCCAGATGGCTGATG	89
SLC17A5 (2)	Solute Carrier Family 17 member 5	F: ACCCCTGAGAACACGATCAG	R: TGTGAAGAAAATGGCACCAA	84
SLC2A3/GLUT3	Solute Carrier Family 2 Member 3	F: TCCCCTCAGCTGCATTCTAT	R: CCAGAAGACAACGAGGAAGC	71
ST3GAL3	ST3 Beta-Galactoside Alpha-2,3-Sialyltransferase 2	F: CTCTGGGGTCACGAATTGAT	R: ACGTCCTTCTCGAAGCCTTT	79
ST3GAL5 (1)	ST3 Beta-Galactoside Alpha-2,3-Sialyltransferase 5	F: GCACCACTGTCTGACCTTGA	R: GCCTGAAGCCAGTTGAAGTC	86
ST3GAL5 (2)	ST3 Beta-Galactoside Alpha-2,3-Sialyltransferase 5	F: AACCAGTTCGACGTGGTGAT	R: CCCCCTCTGGGTAAGTCATC	103
ST8SIA1 (1)	ST8 Alpha-N-Acetyl-Neuraminide Alpha-2,8-Sialyltransferase 1	F: TTTGTCATGCGATGCAATCT	R: ACCTTTGCCGAATTATGCTG	106
ST8SIA1 (2)	ST8 Alpha-N-Acetyl-Neuraminide Alpha-2,8-Sialyltransferase 1	F:GCTGTGGCCGTCAAATAGAT	R:AGCCGTCACCAAATGACTTT	110
SYNPO	Synaptopodin	F: CAGAGGCAGATGTCAACCAA	R: GTTGTGGTGGCCGTTAGAAG	77
SYP	Synaptophysin	F: GTGACCTCTGGCCTCAACAC	R: CTCCTTGAACACGAACCACA	87
TBP	TATA box binding protein	F: ACGTTCGGTTTAGGTTGCAG	R: CAGGAACGCTCTGGAGTTCT	96
TGFB2	Transforming Growth Factor Beta 2	F: GACCCCACATCTCCTGCTAA	R: ATAGGCTGCATCCAAAGCAC	94
UGCG (1)	UDP-Glucose Ceramide Glucosyltransferase	F: GCTGCCGTATGTAGCAGACA	R: CCTTGGATGAGAGGTTCCAA	76
UGCG (2)	UDP-Glucose Ceramide Glucosyltransferase	F: AGAATGCTTCGTTGCCAGTT	R: TATAAACCACGCCAGGCAAT	103
VEGFA	Vascular Endothelial Growth Factor A	F: GAGGCAAGAAAATCCCTGTGG	R: GCAGGAACATTTACACGTCTGC	85
